# Integration of Transcriptional and Post-transcriptional Analysis Revealed the Early Response Mechanism of Sugarcane to Cold Stress

**DOI:** 10.3389/fgene.2020.581993

**Published:** 2021-01-25

**Authors:** Xing Huang, Yongsheng Liang, Baoqing Zhang, Xiupeng Song, Yangrui Li, Zhengqiang Qin, Dewei Li, Rongfa Chen, Zhongfeng Zhou, Yuchi Deng, Jiguang Wei, Jianming Wu

**Affiliations:** ^1^College of Agriculture, Guangxi University, Nanning, China; ^2^Key Laboratory of Sugarcane Biotechnology and Genetic Improvement (Guangxi), Guangxi Key Laboratory of Sugarcane Genetic Improvement, Sugarcane Research Institute, Guangxi Academy of Agricultural Sciences, Ministry of Agriculture, Nanning, China; ^3^Nanning Institute of Agricultural Sciences, Nanning, China

**Keywords:** miRNAs, integrative analysis, cold stress, sugarcane, miRNA targeted genes

## Abstract

Cold stress causes major losses to sugarcane production, yet the precise molecular mechanisms that cause losses due to cold stress are not well-understood. To survey miRNAs and genes involved in cold tolerance, RNA-seq, miRNA-seq, and integration analyses were performed on *Saccharum spontaneum*. Results showed that a total of 118,015 genes and 6,034 of these differentially expressed genes (DEGs) were screened. Protein–protein interaction (PPI) analyses revealed that ABA signaling via protein phosphatase 2Cs was the most important signal transduction pathway and late embryogenesis abundant protein was the hub protein associated with adaptation to cold stress. Furthermore, a total of 856 miRNAs were identified in this study and 109 of them were differentially expressed in sugarcane responding to cold stress. Most importantly, the miRNA–gene regulatory networks suggested the complex post-transcriptional regulation in sugarcane under cold stress, including 10 miRNAs−42 genes, 16 miRNAs−70 genes, and three miRNAs−18 genes in CT vs. LT0.5, CT vs. LT1, and CT0.5 vs. LT1, respectively. Specifically, key regulators from 16 genes encoding laccase were targeted by novel-Chr4C_47059 and Novel-Chr4A_40498, while five *LRR-RLK* genes were targeted by Novel-Chr6B_65233 and Novel-Chr5D_60023, 19 PPR repeat proteins by Novel-Chr5C_57213 and Novel-Chr5D_58065. Our findings suggested that these miRNAs and cell wall-related genes played vital regulatory roles in the responses of sugarcane to cold stress. Overall, the results of this study provide insights into the transcriptional and post-transcriptional regulatory network underlying the responses of sugarcane to cold stress.

## Introduction

Sugarcane planting, yield, and quality are affected by abiotic factors including low-temperature stress. Previous research has shown that the response of sugarcane to cold stress is regulated by sophisticated mechanisms at the morphological, anatomical, physiological, and biochemical levels. Once subjected to cold stress, sugarcane leaves dry up, growth points and lateral buds become frostbitten, and the tilling rate and number of effective perennial stems are affected, especially at the seedling stage. Cold stress markedly inhibits sugarcane seedling growth and survival rates fall (Rasheed et al., 2010; Pang et al., 2015). The thickness of sugarcane leaves, the area of vesicular cells, the thickness of thick-walled vascular bundle tissue, and electrolyte extravasation rates are all known to be positively correlated with cold resistance (Zhu et al., 2019). Indeed, at the seedling stage, the degree of peroxidation of the leaf lipid membrane under cold stress is enhanced and occurs in concert with membrane permeability and relative conductivity. The content of soluble sugar, proline, and 3,4-methylenedioxyamphetamine (MDA) also increases (Ding et al., 2006; Wang et al., 2016a), while chlorophyll content decreases with the decrease in temperature and increased duration of cold temperature treatment. Cold stress also inhibits the activity of ribulose-1,5-diphosphate carboxylase (Rubisco) and phosphoenolpyruvate carboxylase (PEP), while SOD (superoxide dismutase) and POD (peroxidase) increase. Traditional genetic and molecular analyses have identified that COLD1-CRLK1/2-MEKK-MPK-ICE-CBF-CORs and CRPK1-14-3-3-CBF are important cold signal sensing pathways in rice and *Arabidopsis* (Guo et al., 2018; Shi et al., 2018). Recent RNA-seq research has revealed the presence of regulated mechanisms that are more complex in other plants (Li et al., 2016). Almost 50% of paper mulberry transcriptional factors are involved in cold stress responses (Peng et al., 2015). In addition, 1,841 differentially expressed genes (DEGs) are involved in cold stress response in maize (Li et al., 2017). A total of 47 genes are upregulated in wheat, whereas just six are downregulated in the ICE1-CBF-COR pathway as a component of cold stress response. Ten genes in the ABA-dependent pathway are upregulated, while four are downregulated across these four genotypes, confirming that ABA accumulates when plants are exposed to low temperatures (Li et al., 2018).

The regulation of gene expression via microRNA (miRNA) is a negative mechanism that occurs in response to cold stress in plants at post-transcriptional levels. In plants, miRNAs are divided into two groups, conserved, and species-specific (Esposito et al., 2020). In recent years, numerous cold-responsive miRNAs have been identified in different plant species, and some of them are involved in the CBF-dependent pathway, triggering the elimination of ROS, modifying cellular antioxidant capacity, and in the auxin signaling pathway (Megha et al., 2018). miR319 is downregulated by cold stress in rice while the overexpression of miR319 or target RNA interference leads to the upregulation of cold-responsive genes and improved cold tolerance (Thiebaut et al., 2012; Yang et al., 2013; Wang et al., 2014). miR160 and miR167 have been identified as temperature-responsive miRNAs in cotton. The miR160 family also exhibits lower expression levels at 12°C compared with the control at 25°C (Wang et al., 2016b). One hundred and seventy-two miRNAs belonging to 39 families and 126 unique examples were identified in *Nelumbo nucifera* (Zou et al., 2020). 172 miRNA–target pairs have been identified as being involved in plant temperature responses by integrating miRNA with mRNA expression profiles. Three conserved and 25 predicted miRNAs have also been found to be involved in response to cold stress in *Brachypodium* (Zhang et al., 2009).

Prior to the sequencing of the complete sugarcane genome, expressed sequence tags (ESTs) and RNA-seq were both used to determine the mechanisms involved in cold stress response. In one example, 34 cold-inducible ESTs were identified in 2003 from 1,536 ESTs in sugarcane (*Saccharum* sp. cv SP80-3280) exposed to cold stress (Nogueira et al., 2003). Transcriptome profiles from the low temperature-tolerant *S. spontaneum* clone IND 00-1037 are also analyzed, as well as 2,583 genes that are upregulated and 3,302 that are downregulated in a low temperature stress treatment (Dharshini et al., 2016). A total of 170 cold-responsive TFs in 30 families are also differentially regulated (Selvarajan et al., 2018). Transcriptome profiles of *S. spontaneum* under cold stress indicate that certain genes involved in transmembrane transporter activities are absent from cold-susceptible sugarcane (Jong-Won et al., 2015). Additional work on small RNA transcriptomes from sugarcane has also been possible via deep sequencing (Sternes and Moyle, 2015). However, analysis of miRNA–genes under cold stress has not been undertaken since publication of the complete sugarcane genome (Zhang et al., 2018).

The wild relative of sugarcane, *Saccharum spontaneum* L., is known for its adaptability to environmental stressors, particularly cold stress conditions. Thus, to gain a comprehensive understanding of the molecular mechanisms involved in the response of sugarcane seedlings to cold stress, we studied the expression profiles and relevant genes in *S. spontaneum* GX87-16 seedlings treated with cold stress (4°C). In addition, we integrated a DEG and differentially expressed miRNA analysis and constructed a regulation network of miRNA–mRNA. The findings from this study provide insight into the transcriptional and post-transcriptional regulation of cold tolerance and low temperature stress in sugarcane.

## Methods

### Plant Materials and Cold Stress Treatment

Stems of *S. spontaneum* GX87-16 were planted in pots and grown in an artificial incubator at 28°C under a 16-h/8-h (light/ dark) photoperiod. After 2 weeks of growth, 2 h after entering the light culture, seedlings from six pots were transferred to an artificial incubator, which had been set to 4°C for cold stress treatment. Mature leaves were collected from treatment plants at 0.5 h and 1 h after cold treatment and denoted as LT0.5 and LT1. Meanwhile, another three potted plants were sampled at the time when the six pots were transferred to a 4°C artificial incubator; these three repeats were considered as controls (CT), namely, the 0 h of cold stress. Collected leaves were frozen in liquid nitrogen and stored at −80°C prior to RNA extraction.

### Construction and Sequencing of mRNA-Seq and Small RNA Libraries

Total RNA was extracted using the Trizol reagent (Invitrogen), and RNA integrity was assessed using an RNA Nano 6000 Assay Kit in an Agilent Bioanalyzer 2100 system (Agilent Technologies, CA, United States). RNA concentrations were measured using a Qubit® RNA Assay Kit in a Qubit® 2.0 Fluorometer (Life Technologies, CA, United States). Equal amounts of RNA from all samples were used for the construction of mRNA-Seq and small RNA libraries.

A series of mRNA-Seq libraries were prepared using an Illumina TruSeq RNA Sample PrepKit following the manufacturer's protocols. Sequencing was performed via paired-end reads (2 × 151 base pairs, bp) using an Illumina X Ten sequencing platform. Bands of RNA between 18 and 40 nt in length were isolated for small RNA sequencing. Libraries were prepared following the Small RNA Sample Preparation Protocol (Illumina) and sequenced on an Illumina Hiseq-2500 sequencing platform using SE50.

### Processing of mRNA-Seq Data

To preprocess miRNA-Seq data, adaptor sequences were removed alongside low-quality (<Q20) bases at 5′ and 3′ ends using the software Trimmomatic (v0.30) (Bolger et al., 2014). Reads longer than 70 bp were then used for further experiments. These reads were mapped onto the sugarcane genome (Zhang et al., 2018) using the software Bowtie 2 (2.1.0) (Langmead and Salzberg, 2012) with default parameters set after the preprocessing of mRNA-Seq data. The software TopHat2 was then used to perform a sequence comparison between clean reads and the reference genome to obtain positional information for the reference genome or gene and sequence information unique to the sample. Gene expression levels were presented as fragments per kilo base per million reads (FPKM); thus, genes with FPKM values >1 were retained for further analysis.

A false discovery rate (FDR) <0.05 was used to detect DEGs. After processing with the software DEseq, we applied a more than 2-fold change as the criterion to classify DEGs between two pairs (www.bioconductor.org). Resultant *P*-values were then adjusted using Benjamini and Hochberg's approaches for controlling the FDR.

Functional annotations were determined using the Gene Ontology (GO) and Kyoto Encyclopedia of Genes and Genomes (KEGG) pathway enrichment analyses. Default parameters in the software AmiGO were used to obtain GO terms for each gene as well as analyze functional enrichment via hypergeometric tests with FDR correction. This procedure enabled us to obtain an adjusted *p* < 0.01 between test gene groups and the whole annotation dataset. DEGs in the KEGG pathway were analyzed using the software Cytoscape (Ideker, 2011) using the ClueGO plugin (Wouter et al., 2014).

### Processing miRNA-Seq Data

In this step, clean reads were screened from raw data by removing adaptors, poly A sequences, and low-quality bases at both ends of small RNA reads. Reads were then trimmed and cleaned by removing sequences smaller than 18 nt or longer than 40 nt. Values for the sequence duplication level, GC content, Q20, and Q30 in clean data were then calculated. All downstream analyses were based on high quality clean data.

To identify known and novel miRNAs, high-quality clean reads were mapped to the *S. spontaneum* genome using the software Bowtie 2 (2.1.0) (Langmead and Salzberg, 2012) and searched against NCBI, Rfam, and Repbase databases to remove known classes of RNAs (i.e., mRNA, rRNA, tRNA, snRNA, and snoRNA) and repeats. The remaining reads were then used to detect both known and novel (potentially species-specific) miRNAs among the 5,940 mature miRNAs in plants using mirBase Release 19 (Ana and Sam, 2011).

We performed a differential expression analysis under two conditions using the DESeq R package (1.10.1). This approach provides a statistical analysis for determining differential expression in digital miRNA expression data using a model based on the negative binomial distribution. Thus, miRNAs with an adjusted *p* < 0.01 were assigned as differentially expressed.

### Predicting Potential miRNA Targets: A Joint Analysis of miRNA and mRNA

The psRNATarget tool (http://plantgrn.noble.org/psRNATarget/) and software TargetFinder were used to predict *S. spontaneum* mRNA targets of putative miRNAs. The software packages BLAST and KOBAS2.0 were then used to compare and carry out a functional annotation of discovered target genes vs. the NR, Swiss-Prot, GO, COG, KOG, Pfam, and KEGG databases. Finally, miRNA and mRNA were related to one another using their interaction in the STRING protein database (http://string-db.org/).

All clean sequencing data used in this study are available in the NCBI Sequence Read Archive (http://www.ncbi.nlm.nih.gov/sra/) under accession numbers PRJNA636260 for transcriptomics and PRJNA635765 for miRNA-seq data.

### Validation of mRNA and miRNA Relative Expression by QPCR

The RNA used for RNA-seq was used for the QPCR validation of the mRNA expression level. The first-strand cDNA for each sample was prepared from 500 ng of total RNA using SuperScript II reverse transcriptase (Takara, Dalian, China) following the manufacturer's instructions, and template cDNAs were diluted 10-fold for QPCR. Primers were designed using the Primer 5 program and are listed in Supplementary Table 1. Samples and standards were run in triplicate on each plate using the SYBR Premix Ex Taq™ II kit (Takara, Dalian, China) on a StepOne™ real-time PCR system (Applied Biosystems, Foster City, USA) following the manufacturer's recommendations. QPCR was performed in a 20-μL reaction volume containing 6.8 μL of ddH_2_O, 10 μL of SYBR Premix Ex Taq II, 0.4 μL of ROX Reference Dye II, 0.4 μL of forward primer (10 μmol/L), 0.4 μL of reverse primer (10 μmol/L), and 2 μL of template cDNA. The PCR programs were run as follows: 30 s of pre-denaturation at 95°C, 40 cycles of 5 s at 95°C, and 30 s at 60°C, followed by steps for dissociation curve generation. Dissociation curves for each amplicon were carefully examined to confirm the lack of multiple amplicons at different melting temperatures. Relative transcript levels for each sample were obtained using the comparative cycle threshold method using the cycle threshold value of transcript Sspon.005D0014601 encoding a glyceraldehyde-3-phosphate dehydrogenase was used as a control. The 2^−ΔΔ*Ct*^ method was used to calculate the data.

The RNA used for miRNA-Seq was used for the QPCR validation of miRNA expression. No more than 500 ng of each miRNA sample was used for miRNA first-strand preparation according to Stem-loop miRNA cDNA Synthesis kit (EnzyValley, Lot: T435A). Stem-loop primers were listed in Supplementary Table 1. QPCR was performed (Stem-loop miRNA qPCR SYBR kit, Lot: T436A) in a 20-μL reaction volume containing 3.5 μL of ddH_2_O, 10 μL of 2× Stem-loop miRNA qPCR SYBR ProMix, 0.5 μL of ROX Reference Dye II, 0.5 μL of forward primer (10 μmol/L), 0.5 μL of Uni-SL reverse primer (10 μmol/L), and 5 μL of template cDNA. The PCR programs were run as follows: 10 min of pre-denaturation at 95°C, 40 cycles of 5 s at 95°C, and 30 s at 60°C, followed by steps for dissociation curve generation. The 2^−ΔΔ*Ct*^ method was used to calculate the data. The U6 snRNA was taken as the control.

## Results

### Gene Identification and Their Functional Annotation Analysis in This Study

A total of nine RNA-seq libraries from low temperature (LT)-treated and control (CT) seedlings were constructed for high-throughput sequencing. After the removal of low-quality reads and adapter sequences, 346,617,812 clean paired-end reads were obtained from raw data, with between 33,119,4345 and 44,605,663 obtained from each sample (Supplementary Table 2). Nearly 80% of these paired-end reads could be mapped to the reference genome, and 118,015 potential genes were identified with FPKM values between 0 and 89,290.4 (Sspon.007A0027210) (Supplementary Table 3). In total, 34,052 (28.85%), 56,311 (47.71%), 78,517 (66.53%), 80,524 (68.23%), 87,333 (74.00%), 101,433 (85.95%), and 108,262 (91.73%) genes were annotated in the COG, KOG, Swissprot, Pfam, GO, TrEMBL, and nr databases, respectively. Approximately 10.07% (11,887) of genes were annotated in the KEGG database (Supplementary Tables 3, 4, Supplementary Figure 1), with most genes significantly matched with *Sorghum bicolor* (70.32%).

### Identifying Cold-Responsive Genes in Sugarcane

**Figure 1:**
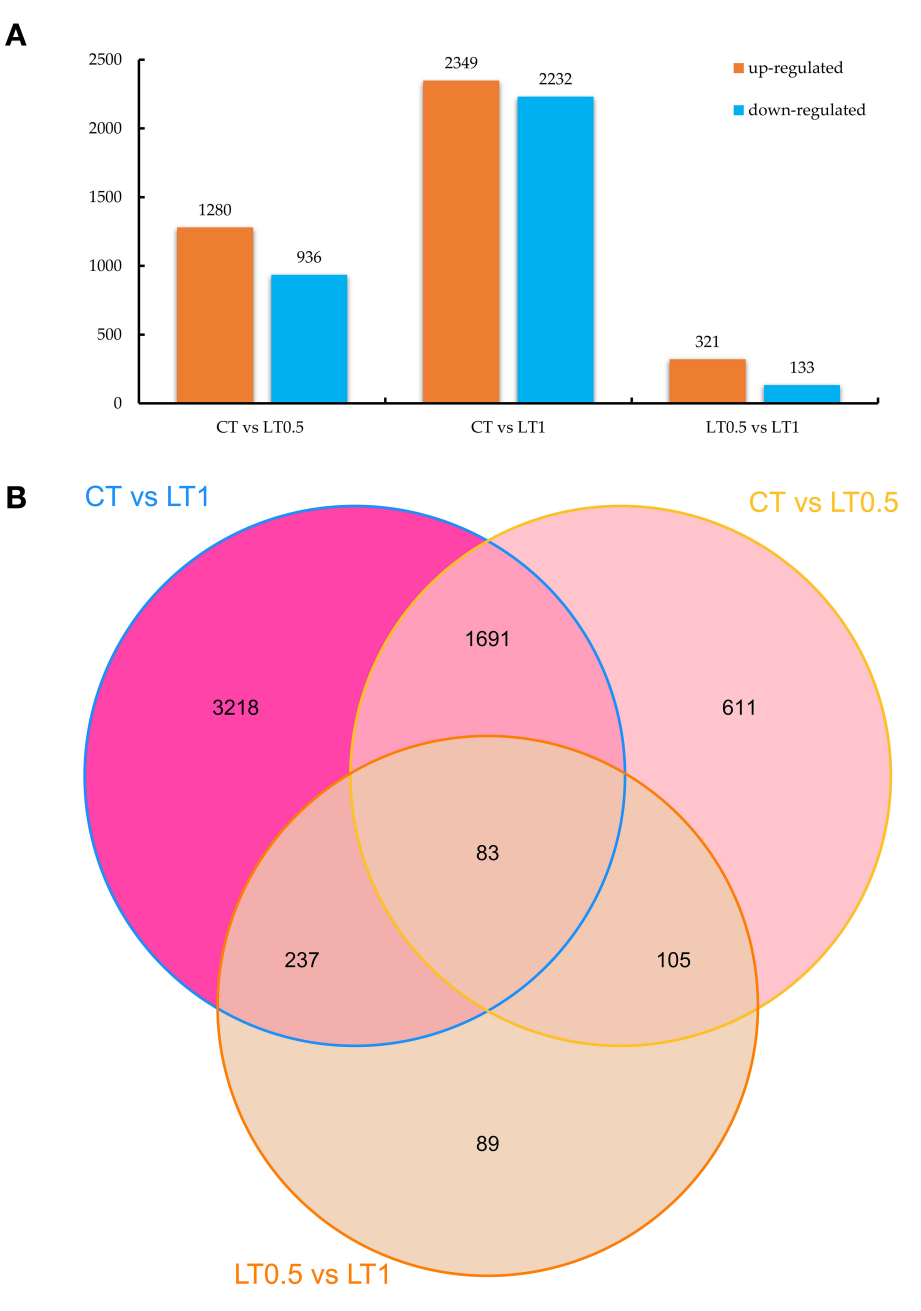
DEG statistics for comparisons. **(A)** Number of downregulated and upregulated DEGs in CT vs. LT0.5, CT vs. LT1, and LT0.5 vs. LT1, respectively. **(B)** Venn diagram of DEGs distributed in every sample.

**Figure 2:**
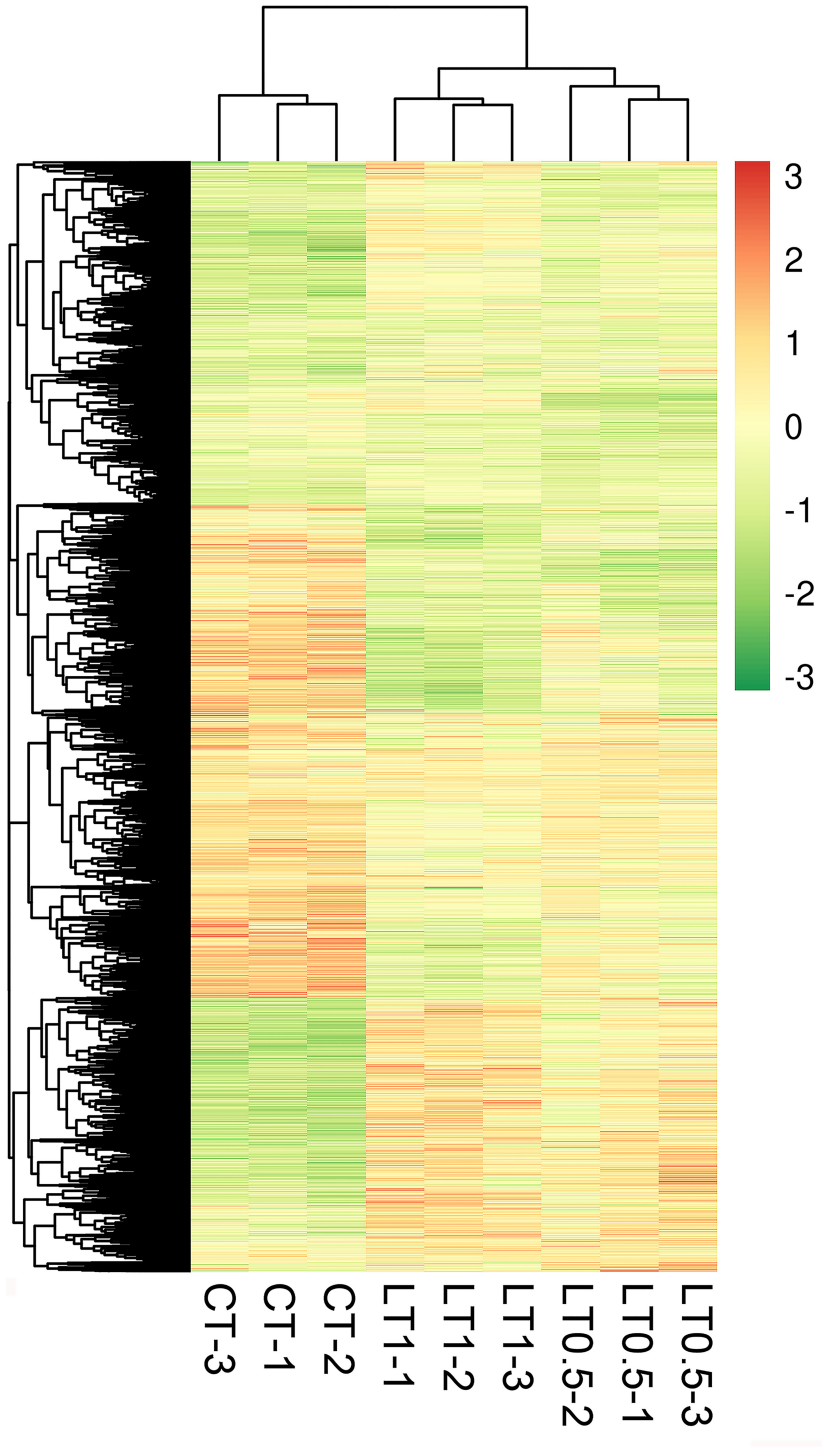
Heat map of *Saccharum spontaneum* genes responding to cold stress at the transcription level. To be considered differentially expressed, a transcript must have fragments per kilobase of transcript per million mapped reads (FPKM) ≥ 0.3 in at least one sample, a 2-fold (or greater) change between samples, and *P* ≤ 0.05. Red indicates high expression, white indicates intermediate expression, and green indicates low expression.

A total of 6,034 DEGs (10.19% of total genes) were detected via DEGseq analysis (*p*-value <0.005 and |log_2_ (fold change)| > 1) under cold stress conditions. Compared with CT, the LT0.5 and LT1 treatments had 2,216 and 4,581 DEGs, respectively, including 1,280 and 936; 2,349 and 2,232 upregulated and downregulated DEGs for each compared pair. When LT1 was compared with LT0.5, 454 genes, including 321 upregulated and 133 downregulated DEGs, were identified (Figure 1A, Supplementary Tables 5–7). The resultant Venn diagram suggests that 83 core DEGs were shared across all compared pairs, and the largest number of unique DEGs was 3,218 in the CT vs. LT1 comparison (Figure 1B). The heat map for all samples indicates that biological repeats reached their expected effect and that four dominant expression profile clades were found via this dynamic hierarchical clustering approach (Figure 2).

**Figure 3:**
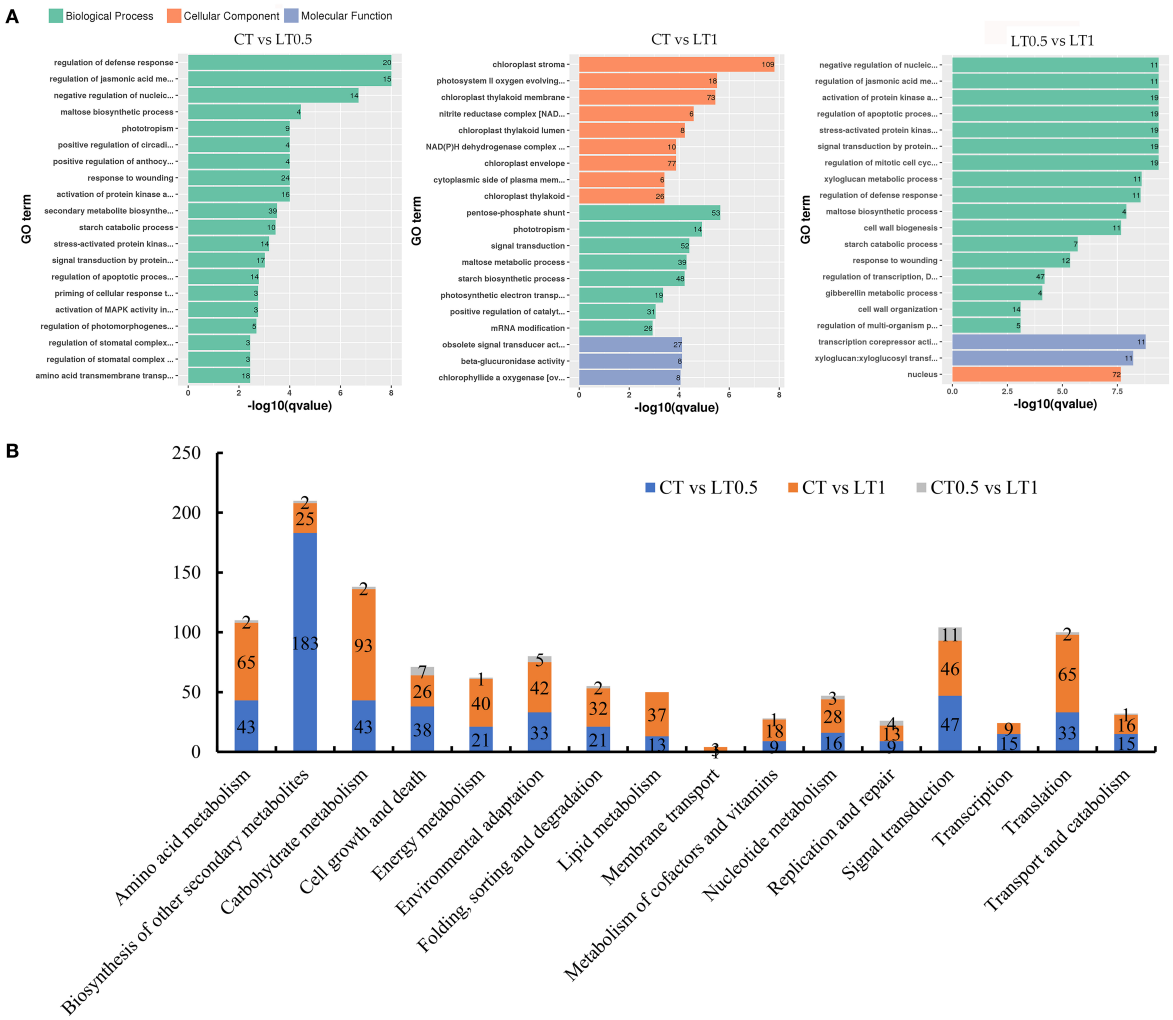
Annotation and functional classification of DEGs. **(A)** Histogram of most enriched Gene Ontology (GO) classifications of *S. spontaneum* DEGs under cold stress. The ordinate is the enriched GO term; the abscissa is the enriched *q* value of the term and the number of differential genes on the column. Different colors are used to distinguish biological processes, cellular components, and molecular functions. **(B)** Statistical analysis of DEGs mapped to KEGG pathways including carbohydrates, energy, and lipid metabolisms.

Functional annotations of all DEGs for each compared pair were presented in Supplementary Tables 5–8 and Supplementary Figure 2. Comparisons between CT and LT0.5 shows that 1,744 DEGs can be assigned to GO terms; of these, the term “regulation of defense response” was the most highly enriched among both upregulated and downregulated DEGs (Figure 3A). In the 1-h cold stress treatment, 3,645 DEGs could be GO annotated and the chloroplast was most affected.

The resultant KEGG pathway classification (Supplementary Table 8 and Figure 3B) reveals that most significantly changed transcripts belong to metabolic pathways (especially those for starch, sucrose, pyruvate, and carbon fixation), signal transduction (plant hormones), environmental adaptation (plant–pathogen interaction), and the biosynthesis of secondary metabolites (mainly related to phenylpropanoid biosynthesis).

### Protein–Protein Interaction Network Analysis in the mRNA Level Under Cold Stress

We constructed an interaction network based on the DEGs retrieved from known PPI databases. In terms of comparisons between CT and LT0.5, 1,186 DEGs can be linked via 1,100 known relationships, including activation, binding, and inhibition (Supplementary Figure 3A). The gene Sspon.004C0004740 can be identified as the core hub encoding an ATP synthase delta subunit, which was downregulated in the LT0.5 treatment. Sspon.006B0008980 (peptidyl-prolyl cis-trans isomerase CYP38), Sspon.008C0004432 (glycosyl hydrolases), Sspon.001D0013480 (fibrillarin), and Sspon.005A0004900 (ribosomal protein L23) are also important candidate DEGs that function in response to a half an hour cold stress.

**Figure 4:**
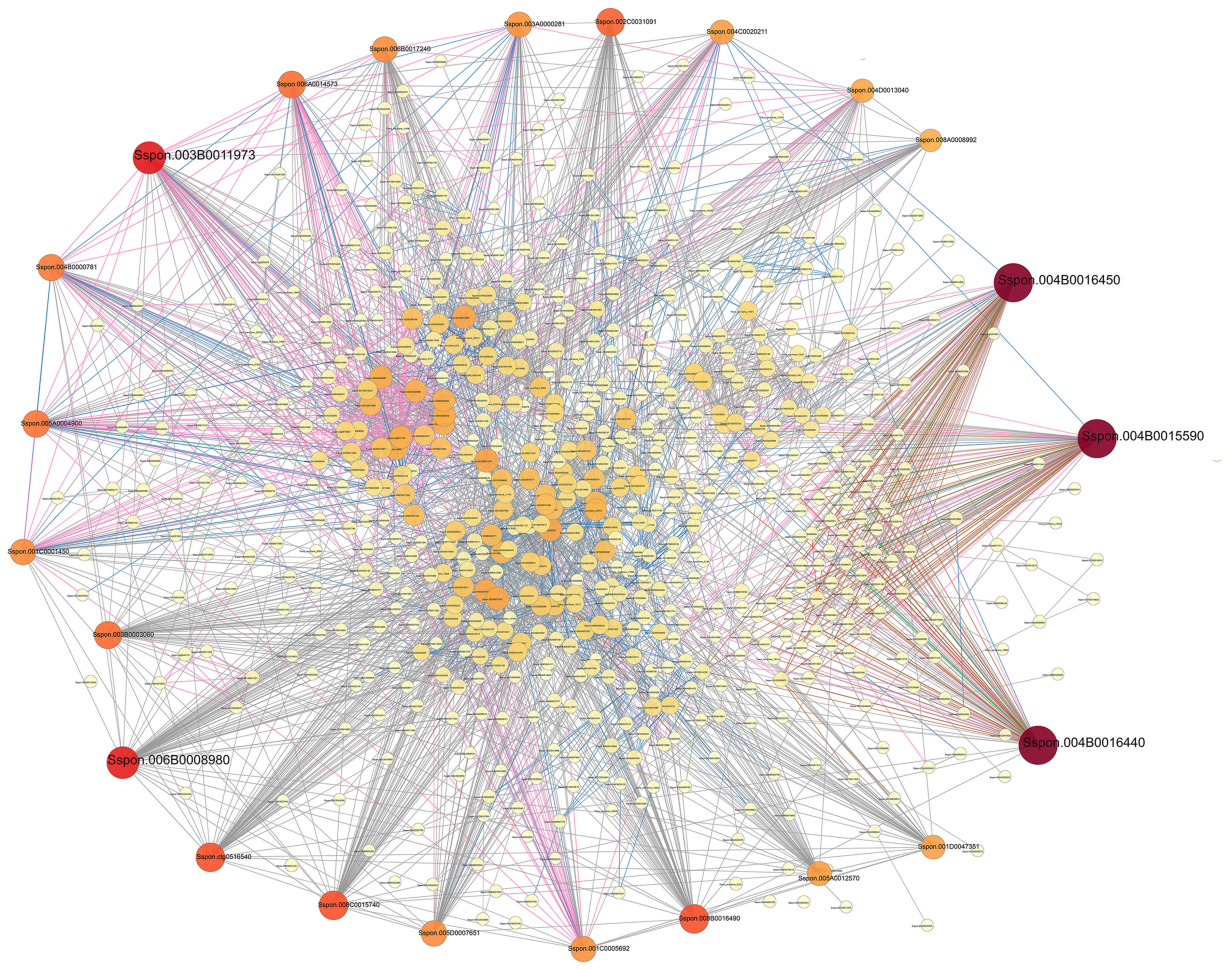
A PPI (protein–protein interaction) network of DEGs from CT vs. LT1. The interaction relationship in the STRING protein interaction database (http://string-db.org/) was then used to analyze the DEG coding protein interaction network. The size of a node in this interaction network is proportional to the degree of this node; thus, the more edges connected to a node, the greater its degree and the larger the node, and the darker the color. Nodes may be in a more central position in the network, and lines of different colors represent varied interactions. The thicker the line, the higher the score value.

After a 1-h cold stress, a total of 2,507 DEGs could be linked via 6,230 known relationships (Figure 4). Significantly, three DEGs (i.e., Sspon.004B0016440, Sspon.004B0016450, and Sspon.004B0015590) encoding the protein phosphatase 2C and the cyclic nucleotide-binding/kinase domain-containing protein (PP2C family) can all be proposed as potential hubs. Similarly, Sspon.003B0011973 (ribosomal protein L13) and Sspon.006B0008980 (peptidyl-prolyl cis-trans isomerase CYP38) also appear to be of importance to the PP2C protein family.

In terms of comparisons between CT0.5 and LT1, 230 DEGs could be linked via 1,600 known relationships (Supplementary Figure 3B). Four TIFY family proteins, including Sspon.001B0025400, Sspon.001D0021420, Sspon.001B0025420, and Sspon.001C0021660, were calculated as the core regulated protein that interacted with other genes through 13 linkages. TIFY is a plant-specific family involved in the regulation of plant-specific biologic processes, stress, and responses to phytohormones.

### Conserved and Novel miRNAs Identified in Sugarcane in This Study

To identify the miRNAs that respond to cold stress, we constructed nine structural RNA (sRNA) libraries from nine samples. These libraries yielded 110,555,326 clean reads by high-throughput sequencing, which was ~11.05 million clean reads per library (Supplementary Table 9). Mapped sRNAs were then annotated and classified into sRNA, known miRNAs, precursor RNAs, intergenic RNAs, and unknown sRNAs. To verify these results, we analyzed Pearson correlation coefficients of the miRNA expression between biological duplicates (Supplementary Figure 4); all the Pearson values for miRNA expression were >0.82.

**Figure 5:**
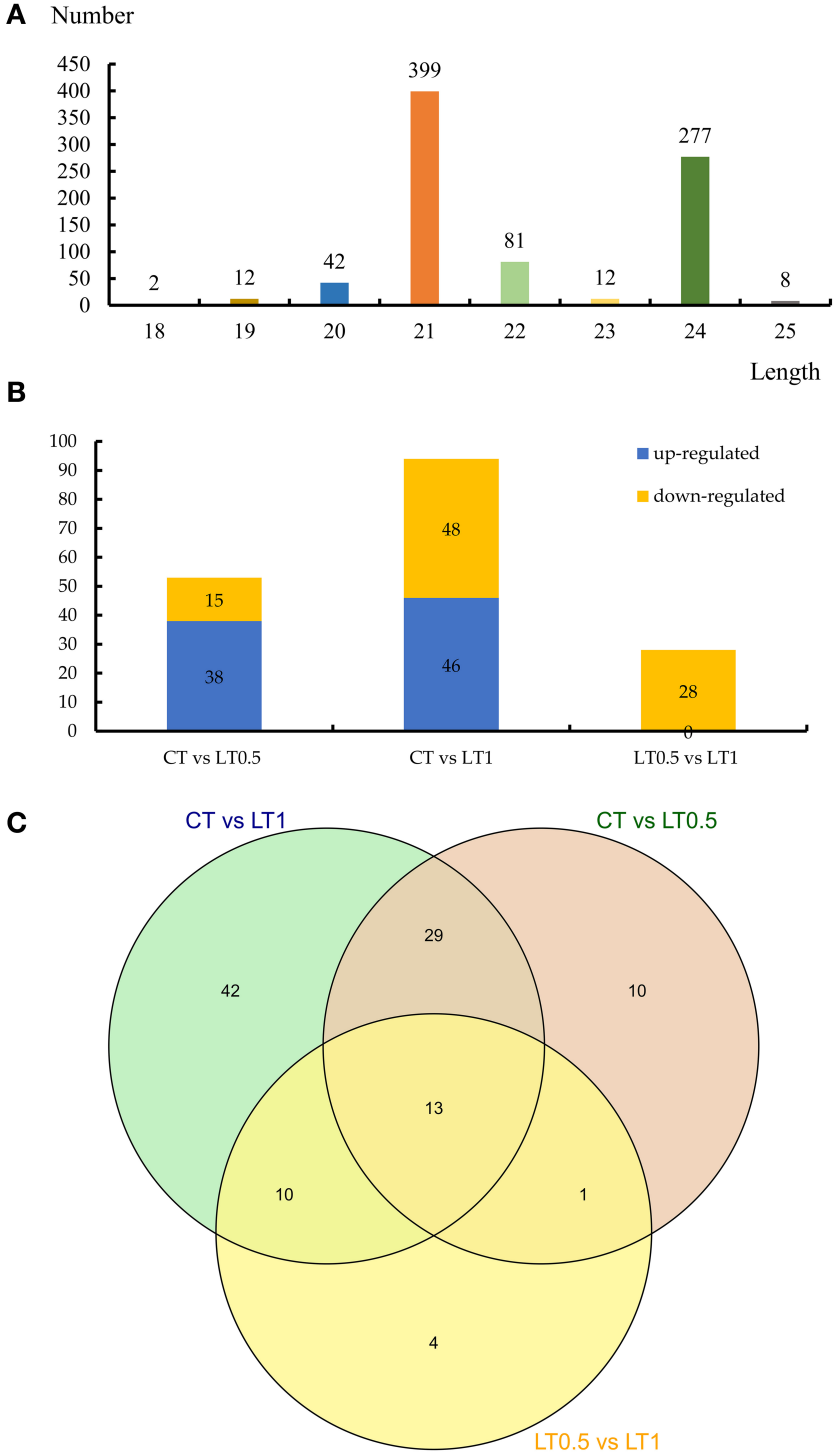
Statistics of expressed miRNAs in this study. **(A)** The length distribution of miRNAs identified. **(B)** Number of downregulated and upregulated differentially expressed miRNAs in CT vs. LT0.5, CT vs. LT1, and LT0.5 vs. LT1. **(C)** Venn diagram of differentially expressed miRNAs distributed in samples.

We then obtained 20 conserved and 836 novel miRNAs belonging to 13 families from nine libraries (Supplementary Tables 10, 11). Expression profiles of identified miRNAs reveal great variation in TPM values, ranging from 83,065.85 to <10. Four miRNAs (Novel-Chr4B_41904, Novel-Chr4C_44656, Novel-Chr4C_44658, and Novel-tig00009572_93271) belong to the miR396 family and were highly expressed in all samples. Almost of all the identified miRNAs (835 of 856) were shared by all samples, indicating that these were stable in *S. spontaneum*, even under cold stress. sRNAs range from 18 nucleotides (nt) to 30 nt long, while the majority of mature miRNAs are 21 nt in plants. The distribution of the length of miRNA in *S. spontaneum* showed that 399 miRNAs were 21 nt long and 277 were 24 nt long (Figure 5A). All 16 known miRNAs were 21 nt long, and the first nucleotide was always U, which is different from the length of novel miRNAs, which were 24 nt long (Supplementary Figure 5).

### Prediction of Targeted mRNAs in Sugarcane

A total of 496 genes were targeted by 423 miRNAs (Supplementary Tables 12, 13). A total of 15 known and 290 novel miRNAs corresponded to 65 and 318 targeted genes, respectively. The miRNA Novel-Chr5B_53523 has the largest number of targeted genes 27. A total of 453 targeted genes (accounting for 91.33%) were annotated in KEGG, GO, and other databases (Supplementary Tables 14–16). In the GO annotation, regulation of transcription (35), oxidation–reduction process (34), lignin catabolic process (14), auxin-activated signaling pathway (12), lignin biosynthesis (11), methylation (11), and cell differentiation processes (10) were enriched in biological processes. The nucleus (60), integral components of membrane (46), apoplast (15), cytoplasm (9), and plasma membrane (8) were enriched in the cellular component, and DNA binding (37), ATP binding (24), copper ion binding (14), oxygen oxidoreductase activity (14), oxidoreductase activity and oxidizing metal ions (14), iron ion binding (13), heme binding (12), lipid binding (11), DNA-binding transcription factor activity (10), O-methyltransferase activity (10), protein dimerization activity (10), and zinc ion binding (10) were the top enriched go terms for molecular function. A total of 251 genes were mapped to 132 pathways within the KEGG database; plant hormone signal transduction (29), stilbenoid diarylheptanoid and gingerol biosynthesis (12), and plant–pathogen interactions (11) were the top three annotated pathways (Supplementary Table 16).

### Differentially Expressed miRNAs and Their Targets Under Cold Stress

A total 109 differentially expressed miRNAs exhibiting a more than 2-fold change were identified in *S. spontaneum* after cold stress, including four known and 105 novel miRNAs, respectively (Supplementary Tables 17–19). Compared with the control, the numbers of differentially expressed miRNAs after cold stress treatment for 0.5 h and 1 h were 53 (38 upregulated and 15 downregulated) and 94 (46 upregulated and 48 downregulated). When LT1 was compared with LT0.5, 28 downregulated miRNAs were identified (Figure 5B). The Venn diagram from this analysis suggests the presence of 13 core miRNAs shared by all compared pairs, including 42 unique miRNAs to CT vs. LT1, the largest number (Figure 5C).

TargetFinder was used to predict candidate targets of differentially expressed miRNAs (Supplementary Tables 20–22). In a comparison of CT and LT0.5, 10 miRNAs targeting 42 genes were identified; of these genes, 14 were targeted by miRNAs Novel-tig00084004_92871, referring to signal transduction, transcription, and energy production, such as MYB and LRR-RLK (leucine-rich repeat receptor-like protein kinase). Besides, there were three LRR-RLK genes (Sspon.006A0015270, Sspon.006B0014350, Sspon.003D0010720, and Sspon.005B0004571) were targeted by miRNA Novel-Chr6B_65233, indicating an important function in the regulation of cold stress signal transduction. Furthermore, a total of four laccase genes (Sspon.003D0006520, Sspon.003B0007420, Sspon.003C0020690, and Sspon.003C0010170) were targeted by Novel-Chr4A_40498. These 18 miRNAs were upregulated by cold stress.

A total of 16 miRNAs targeting 70 genes were identified from the comparison of LT1 and CT. The targeted genes include 14 PPR repeat family members (all targeted by Novel-Chr5C_57213), five LRR-RLKs, six scarecrow-like proteins, and four laccases.

Three miRNAs targeting 18 genes were identified in the comparison between CT0.5 and LT1. Among these, total of 11 genes encoding laccase were targeted by Novel-Chr4C_47059 which was downregulated by cold stress.

### Validation of Differentially Expressed mRNAs and miRNAs by Real-time QPCR Analysis

**Figure 6:**
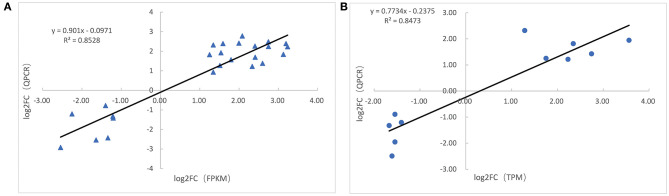
Correlation analysis of validation. **(A)** Correlation analysis of gene expression between qPCR and RNA-seq data. **(B)** Correlation analysis of miRNA expression between qPCR and miRNA-seq data. The X-axis represents RNA-seq (miRNA-seq) data, and the Y-axis represents the qPCR data.

The expression of 10 cold-responsive miRNAs and 25 DEGs was detected by QPCR. The comparisons between QPCR and mRNA-seq (or miRNA-seq) provided in Supplementary Figure 6 suggested that the result of mRNA-seq and miRNA-seq was reliable. This was also confirmed by calculating their Pearson correlation coefficients which were 0.847 and 0.852, respectively (Figure 6).

## Discussion

Cold stress is a major abiotic environmental factor that negatively affects plants by decelerating seed germination, root development, chlorophyll synthesis, and photosynthesis. Considering this, several protective mechanisms are employed by plants to manage adverse conditions. In sugarcane fields in low latitude and complex mountain and plain environments, cold damage has a serious impact on normal sucrose synthesis. To improve cold resistance in sugarcane, genetics and breeding research has emphasized high-quality cold-resistant varieties. A total of 6,034 DEGs were identified in this analysis as responsive to cold stress (4°C). This number is similar to that of previous reports, which proposed the presence of 2,583 upregulated and 3,302 downregulated genes from a low-temperature (10°C) treatment of *S. spontaneum* clone IND 00-1037 (Dharshini et al., 2016).

### Transcriptional Regulatory Network Underlying Cold Stress at the Seedling Stage

Cold sensing occurs via membrane fluidity, calcium, and lipid signaling genes as well as via MAP kinases and phytohormone signaling (Zhao et al., 2017). Over the past several decades, various reports have confirmed that many transcription factors, including CBF, MYB, CAMAT, and NAC, are either directly or indirectly involved in cold stress tolerance; these were crossed with kinase, hormones, circadian cycles, Ca^2+^, and light to accomplish cold signal transduction (Peng et al., 2015; Li et al., 2018; Shi et al., 2018). A total of 4% of upregulated genes were related to signaling, while MYB was downregulated 2.4-fold (Dharshini et al., 2016). In maize seedlings under various stress conditions, 43 TF families containing 403 differentially expressed TFs belong to the ERF, MYB, bZIP, bHLH, WRKY, and NAC TF families (Li et al., 2017). In our study, we found that 56 DEGs belong to MYB, bZIP, GATA, and MADS-box TFs in the CT vs. LT0.5 comparison, while 179 other DEGs related to PP2C, CBL-IPK, CDPK, AMPK, calcium, cAMP, MAPK, phosphatidylinositols, plant hormones, and sphingolipids were the top enriched signaling pathways. These results emphasized that TFs, kinase, and plant hormones played vital roles in the cold signal transduction of *S. spontaneum*.

A total of 14 PP2Cs and 16 PP2Cs in LT0.5 and LT1 compared with CT, respectively, were either downregulated or upregulated under cold stress conditions. Three DEGs (Sspon.004B0016440, Sspon.004B0016450, and Sspon.004B0015590) encoding PP2C proteins are predicted to be potential hubs via string analysis (Figure 4). The first plant PP2C protein (ABI1) was identified as a negative regulator of ABA signaling (Schweighofer et al., 2004). An increasing number of PP2Cs have been defined in different species, and their functions have been subsequently studied. Thus, PP2C proteins were thought to be core members involved in the ABA signaling pathway (Zhu, 2016); however, more recent research showed that the PYL-PP2C-SnRK2 core ABA signaling module activates a MAPK cascade comprised of MAP3Ks MAP3K17/18, MAP2K MKK3, and MAPKs MPK1/2/7/14. These genes might regulate a range of ABA effector proteins via phosphorylation (De Zelicourt et al., 2016) and may also play major roles in the regulation of cell growth as well as cellular biotic and abiotic stress signaling (Lammers and Lavi, 2007; Fan et al., 2019). Proteins belonging to MAPK cascades have also been shown to be involved in the response of plants to cold stress (Markus et al., 2004; Tak et al., 2019), while PTP1 is known to downregulate cold stress (Liu et al., 2016). Thus, we propose that the ABA signaling pathway via PP2C is most important in terms of the cold stress responses of *S. spontaneum* at the seedling stage; however, future research should be conducted on this pathway.

Research has shown that both the regeneration rate and carboxylation efficiency of RuBP decline under chilling stress (Hardigan et al., 2016; Jin et al., 2017). DEG pathways shown to be enriched in GO and KEGG analyses in CT vs. LT1 have emphasized carbohydrate metabolism including how chloroplasts are related to the photosystem and starch and sucrose biosynthesis. These could also be verified by the functional annotation and PPI analysis (Supplementary Figure 3).

Plant secondary metabolism is closely related to abiotic stress responses (Zhang et al., 2017b). Flavonoids play a role in the response of higher plants to abiotic stressors (Jin et al., 2017). For example, in *E. nutans*, seven genes involved in phenylpropanoid biosynthesis are known to be specifically related to cold stress response (Fu et al., 2016). In terms of enzymes involved in phenylpropanoid biosynthesis, phenylalanine ammonia lyase (PAL) is one of the most relevant (Vogt, 2010). The RNA-seq data presented here show that the number of DEGs related to phenylpropanoid pathways accounts for the largest proportion of secondary metabolic pathways. Therefore, we inferred that lignin might play a key adaptive role in cold stress response.

### Cold Stress-Responsive miRNAs: Sugarcane Regulatory Network

Yang et al. (2017) found that 4,002 targets were predicted for 259 miRNAs, which may be due to the absence of a sugarcane genome leading to inaccuracies in prediction. Using the published sugarcane genome as our starting point, we found 365 genes from 305 miRNAs, which is similar to reports on *Astragalus membranaceus* (Abla et al., 2019), *Nelumbo nucifera* (Zou et al., 2020), potato tubers (Ou et al., 2014), sweet potato (Xie et al., 2017), and other plants (Gupta et al., 2014).

Several previous studies on various plant species have confirmed the involvement of miRNAs in cold stress responses (Tang and Chu, 2017; Zeng et al., 2018; Song et al., 2019; Sun et al., 2019; Esposito et al., 2020). A total of 412 sugarcane miRNAs have been isolated from two cultivars, including 261 known and 151 novel miRNAs. These include nodes for ROC22 (relative cold sensitivity) and FN39 (relative cold tolerance); data indicate that 62 of these miRNAs exhibit significant cold stress-induced or depressed expression (Yang et al., 2017), which is less than that found in our study. We found that 109 miRNAs belonging to 11 families responded to cold stress, while miR444 had the most differentially expressed miRNAs (52) followed by miR169-1 (18) (22) and miR396 (13). Compared to previous studies, we also found that miR156, miR168, and miR408 are upregulated under cold stress in sugarcane (Yang et al., 2017). The miR444 in rice has been reported as a key factor relaying antiviral signaling from virus infections to *OsRDR1* expression (Wang et al., 2016a) as well as the promotion of BR biosynthesis (Jiao et al., 2019). This gene is also involved in the nitrate signaling pathway (Yan et al., 2014). Increased expression of miR408 could also lead to improved *Arabidopsis* tolerance to cold and oxidative stress, as shown by a lower ROS level and the induction of genes related to antioxidative function (Ma et al., 2015).

We found 28, 44, and 13 miR444s in CT vs. LT0.5, CT vs. LT1, and CT0.5 vs. LT1, respectively. This is especially the case for Novel-Chr5C_55773, which exhibited induced expression after a 1-h cold stress and targeted three DEGs encoding the apyrase 3 family (Sspon.005D0023491, Sspon.005B0020100, and Sspon.005C0019251). It has been shown that these can maintain plasma membrane integrity and reduce electrolyte leakage under cold stress (Deng et al., 2015). Four LRR-RLKs were also targeted by miR444 (Novel-Chr6B_65223) and miR168 (Novel-Chr5D_60023) after a 1-h cold stress. We propose that at an early stage, cold stress reduces plasma membrane integrity by decreasing the expression of genes encoding apyrase 3 through the regulation of miR444, which also acts as membrane location receptors. These four LRR-RLK genes play an important function in cold stress signal transduction.

An integrated miRNA–gene/mRNA regulation network suggests that 25 miRNAs belong to nine families that play important roles in response to cold stress. Among these nine families, Novel-tig00084004_47811 (miR169), Novel-Chr4C_24049 (miR396), Novel-Chr5C_29143 (miR396), and Novel-tig00005469_46467 (miR159) were significant regulators. Specifically, miR169 and miR396 have been reported to play an indirect role in cold stress response by regulating the TFs (Wang et al., 2017; Sombir et al., 2020). The OsGRF4 transcription factor is targeted by miRNA396, whereas mutations disturb the *GRF4*-miR396 stress response network and results in the development of enlarged grains and cold tolerance enhancements in rice (Chen et al., 2019). Among 14 targets, two genes encoding MYB TFs are thought to be involved in transcriptional regulation. LRR-RLKs and phosphatidylinositol-4-phosphate 5-kinase have also been identified as regulators that function in early cold stress signal transduction.

Gene miR396 in *Poncirus trifoliata* functions in cold tolerance by regulating acc oxidase gene expression and modulating ethylene-polyamine homeostasis (Zhang et al., 2016). This gene also acts as a stress-responsive regulator by conferring tolerance to abiotic stressors and susceptibility to mold infection in tobacco (Chen et al., 2015). A total of 17 genes that encode pentatricopeptide repeat (PPR) family proteins were targeted by Novel-Chr5C_29143 (miR396); these genes play critical roles in all aspects of organelle RNA metabolism including development and stress defense (Zhang et al., 2017a). Another miR396 family member, Novel-Chr4C_24049, was upregulated after a 0.5-h cold stress and is thought to regulate 11 laccases−13 genes, especially Sspon.003B0004220, Sspon.003A0003410, and Sspon.007A0025870. These 11 laccases−13 genes are also differentially expressed under cold stress. Our results suggested that MiR444, miR169, and miR396 play key roles in the regulation of signal transduction, transcriptional, and lignin metabolism in sugarcane responding to cold stress.

## Conclusions

The results of this study show that the multiple regulated mechanisms are involved in the response of sugarcane to cold stress. The signaling pathway mediated by PP2C and LRR-RLK is thought to play a key role in the response of sugarcane to cold stress and has not been previously reported. Most importantly, genes involved in the biosynthesis of polyunsaturated fatty acids and LEA protein, lignin, and pectin pathways were found to be directly involved in cold stress responses targeted by miR444, miR169, and miR396. These key candidate genes and miRNAs will be useful for future research on cold stress response mechanisms. This study provides new insight into the regulatory network at transcriptional and post-transcriptional levels, thus allowing us to better understand the response of sugarcane to cold stress.

## Data Availability Statement

The datasets presented in this study can be found in online repositories. The names of the repository/repositories and accession number(s) can be found in the article/Supplementary Material.

## Author Contributions

YLia, XS, and BZ: data curation. BZ, ZQ, and DL: formal analysis. RC, ZZ, and YD: investigation. YLia and JWu: funding acquisition. JWe and JWu: project administration. XH and JWu: writing—original draft. XH, YLi, and XS: writing—review and editing. All authors approved the final manuscript.

## Conflict of Interest

The authors declare that the research was conducted in the absence of any commercial or financial relationships that could be construed as a potential conflict of interest.

## Supplementary Material

Supplementary Figure 1 Functional annotation of all genes isolated from *S. spontaneum* in this study. **(A)** Statistics for the functional annotation for all identified genes. **(B)** GO assignment for all identified genes. **(C)** Statistics of the NR annotation results mapped to other plants. **(D)** Unigenes were aligned to the COG (Clusters of Orthologous Groups) database to predict and categorize possible functions.

Supplementary Figure 2 Annotation statistics for DEGs in the second level of the GO database **(A–C)** and Annotation statistics for functional categories for DEGs from the COG database **(D,E)**. **(A,D)** Statistics of DEGs from CT vs. LT0.5. **(B,E)** Statistics of DEGs from CT vs. LT1. **(C,F)** Statistics of DEGs from CT0.5 vs. LT1.

Supplementary Figure 3 DEG PPI network. Interaction relationship in the STRING protein interaction database (http://string-db.org/) to analyze DEGs coding in the protein interaction network. **(A)** PPI map for CT vs. LT0.5. **(B)** Map of PPI for CT 0.5 vs. LT1. The size of a node in this interaction network is proportional to the degree of this node; thus, the more edges connected to a node, the greater its degree, the larger the node, and the darker the color. Nodes may be in a more central position in the network and lines of different colors represent varied interactions. The thicker the line, the higher the score.

Supplementary Figure 4 Analysis of miRNA expression correlation among samples and differential expression clustering. **(A)** Clusters of expression patterns of differentially expressed miRNAs. The abscissa represents the sample name and the clustering result of the sample, while the ordinate represents differential miRNA and miRNA clustering results. Different columns represent different samples, while different rows represent different miRNAs. The color in this Figure represents miRNA expression level log2 (TPM) in the sample after normalization (scale number). **(B)** Correlation heat map of the expression levels of two samples. Pearson's Correlation Coefficient was used as an evaluation index for biological repeat correlations. The closer *r* is to 1, the stronger the correlation between the two replicate samples.

Supplementary Figure 5 Base preferences for miRNA. **(A)** Base bias of known miRNAs. **(B)** Base bias of novel miRNAs. The abscissa is the base position of miRNA while the ordinate is the percentage of bases A/U/C/G appearing in the miRNA at that position.

Supplementary Figure 6 QPCR validation for mRNA and miRNA. **(A–C)** The validation of mRNA in CT vs. LT0.5, CT vs. LT1, and LT0.5 vs. LT1. **(D–F)** The validation of miRNA in CT vs. LT0.5, CT vs. LT1, and LT0.5 vs. LT1. The Y-axis represents the value of relative expression which is conversed by Log_2_ (Fold change), the X-axis represents the ID of mRNA or miRNA.

Supplementary Table 1 List of primers used for QPCR validation.

Supplementary Table 2 Statistics of mRNA-seq data and the comparison between sequencing data and reference genome.

Supplementary Table 3 The FPKM value and Functional annotation of all identified genes from *S. spontaneum* in this study.

Supplementary Table 4 Statistics of functional annotation for all identified genes in this study.

Supplementary Table 5 List of DEGs in comparison between CT and LT0.5.

Supplementary Table 6 List of DEGs in comparison between CT and LT1.

Supplementary Table 7 List of DEGs in comparison between LT0.5 and LT1.

Supplementary Table 8 Statistics of KEGG annotation in comparisons.

Supplementary Table 9 Statistics of sRNA-seq reads.

Supplementary Table 10 The family and TPM value of every miRNA in each sample.

Supplementary Table 11 Statistics of miRNAs in each sample.

Supplementary Table 12 Statistics of miRNA with target and target genes.

Supplementary Table 13 List of target genes for every miRNA.

Supplementary Table 14 The function annotation of target genes.

Supplementary Table 15 List of annotation for target genes against GO database.

Supplementary Table 16 List of annotation for target genes against KEGG database.

Supplementary Table 17 The differentially expressed miRNAs in comparison of CT vs. LT 0.5.

Supplementary Table 18 The differentially expressed miRNAs in comparison of CT vs. LT1.

Supplementary Table 19 The differentially expressed miRNAs in comparison of LT 0.5 vs. LT 1.

Supplementary Table 20 Differentially expressed miRNAs under cold stress and their TPM value.

Supplementary Table 21 Corresponding miRNA-mRNA and the functional annotation of mRNA from CT vs. LT0.5.

Supplementary Table 22 Corresponding miRNA-mRNA and the functional annotation of mRNA from CT vs. LT1.

## Abbreviations

MEKK: mitogen-activated protein/ERK kinase kinases
MPK: MAPK phosphatase
CBF: C-repeat cis-element binding factor
COR: cold-regulated
CRPK: calcium-regulated protein kinase
GSH: glutathione
ROS: reactive oxygen species
ERF: ethylene response factor
ARF: auxin response factors
ABA: abscisic acid
SOD: superoxide dismutase
MDA: malondialdehyde
CT: control
LT: low temperature
TF: transcriptional factor
ESTs: expressed sequence tags
FPKM: number of fragments per kilobase of transcript sequence per million base pairs sequenced
COG: clusters of orthologous groups of proteins
PP2C: protein phosphatase 2C
KOG: eukaryotic ortholog groups
KEGG: Kyoto Encyclopedia of Genes and Genomes
GO: gene ontology
DEG: differentially expressed gene
LRR-RLK: leucine rich repeats-receptor-like protein kinase
LEA: late embryogenesis abundant protein
TPM: transcripts per million.
