# Supplementary figures and images for: Integration of Transcriptional and Post-transcriptional Analysis Revealed the Early Response Mechanism of Sugarcane to Cold Stress
Source: Front Genet. 2021 Jan 25;11:581993. doi: 10.3389/fgene.2020.581993 (PMC7868625; doi:10.3389/fgene.2020.581993)

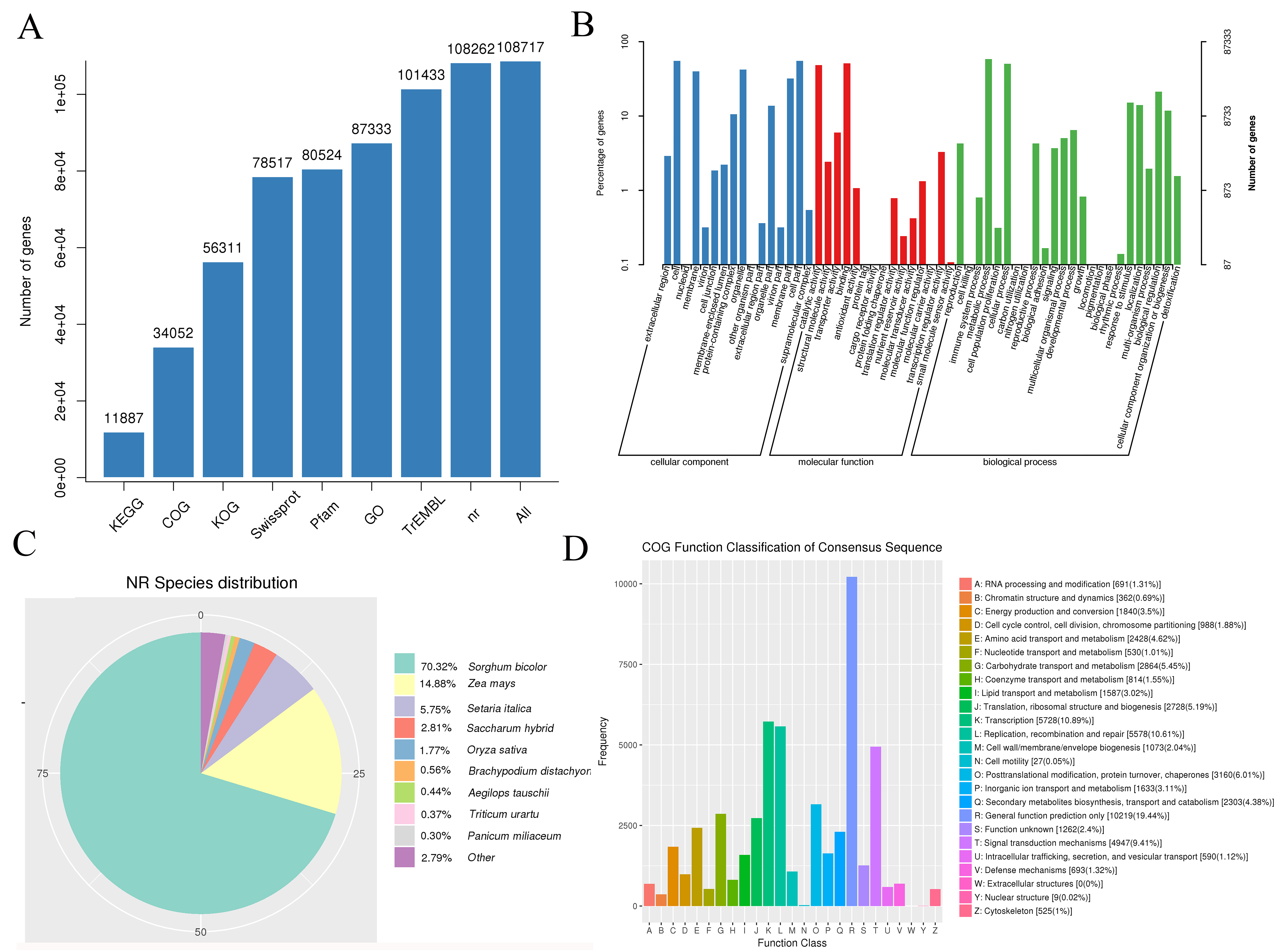

Supplement: Supplementary Figure 1 — Functional annotation of all genes isolated from S. spontaneum in this study. (A) Statistics for the functional annotation for all identified genes. (B) GO assignment for all identified genes. (C) Statistics of the NR annotation results mapped to other plants. (D) Unigenes were aligned to the COG (Clusters of Orthologous Groups) database to predict and categorize possible functions. [file Image_1.TIF]

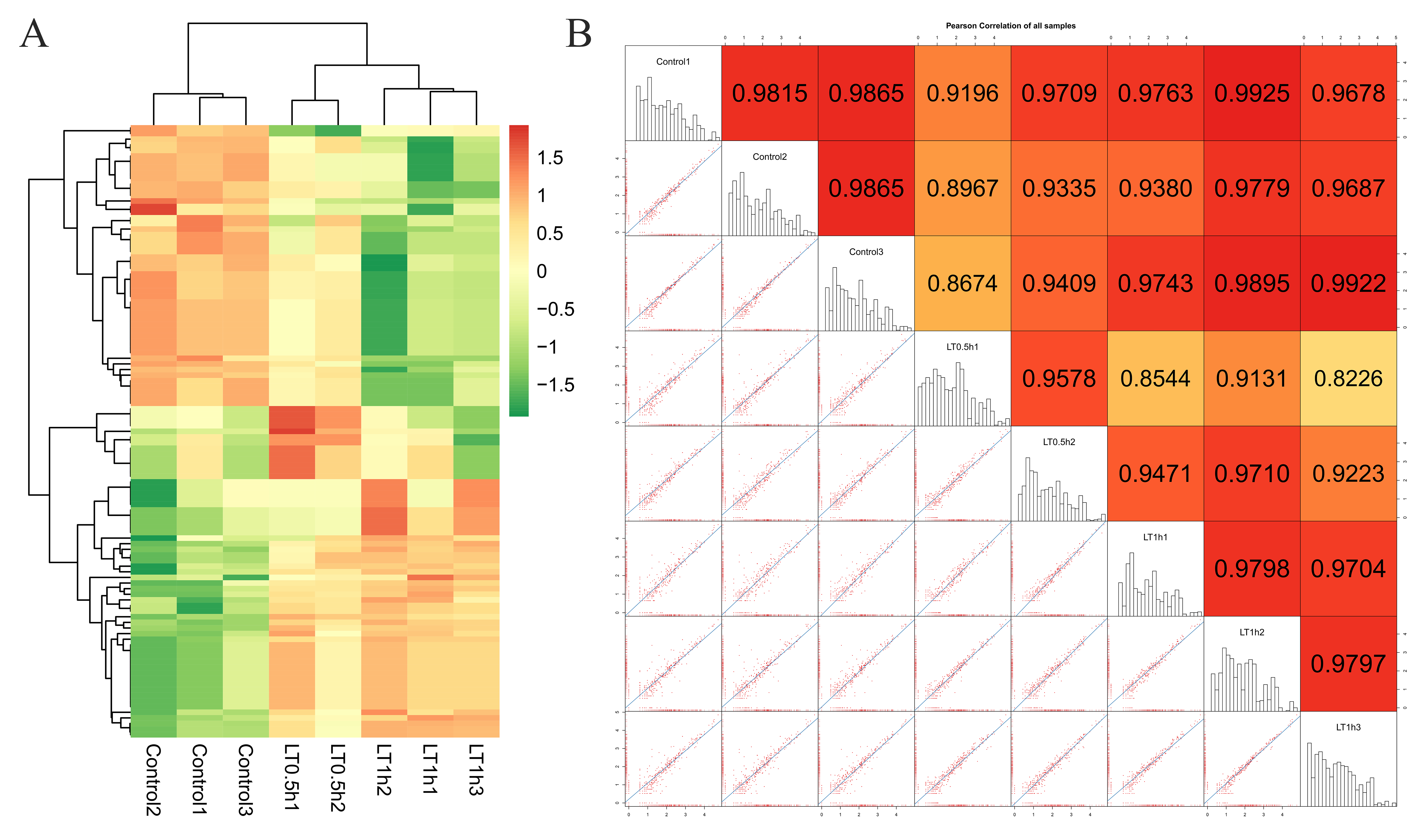

Supplement: Supplementary Figure 4 — Analysis of miRNA expression correlation among samples and differential expression clustering. (A) Clusters of expression patterns of differentially expressed miRNAs. The abscissa represents the sample name and the clustering result of the sample, while the ordinate represents differential miRNA and miRNA clustering results. Different columns represent different samples, while different rows represent different miRNAs. The color in this Figure represents miRNA expression level log2 (TPM) in the sample after normalization (scale number). (B) Correlation heat map of the expression levels of two samples. Pearson's Correlation Coefficient was used as an evaluation index for biological repeat correlations. The closer r is to 1, the stronger the correlation between the two replicate samples. [file Image_4.TIF]

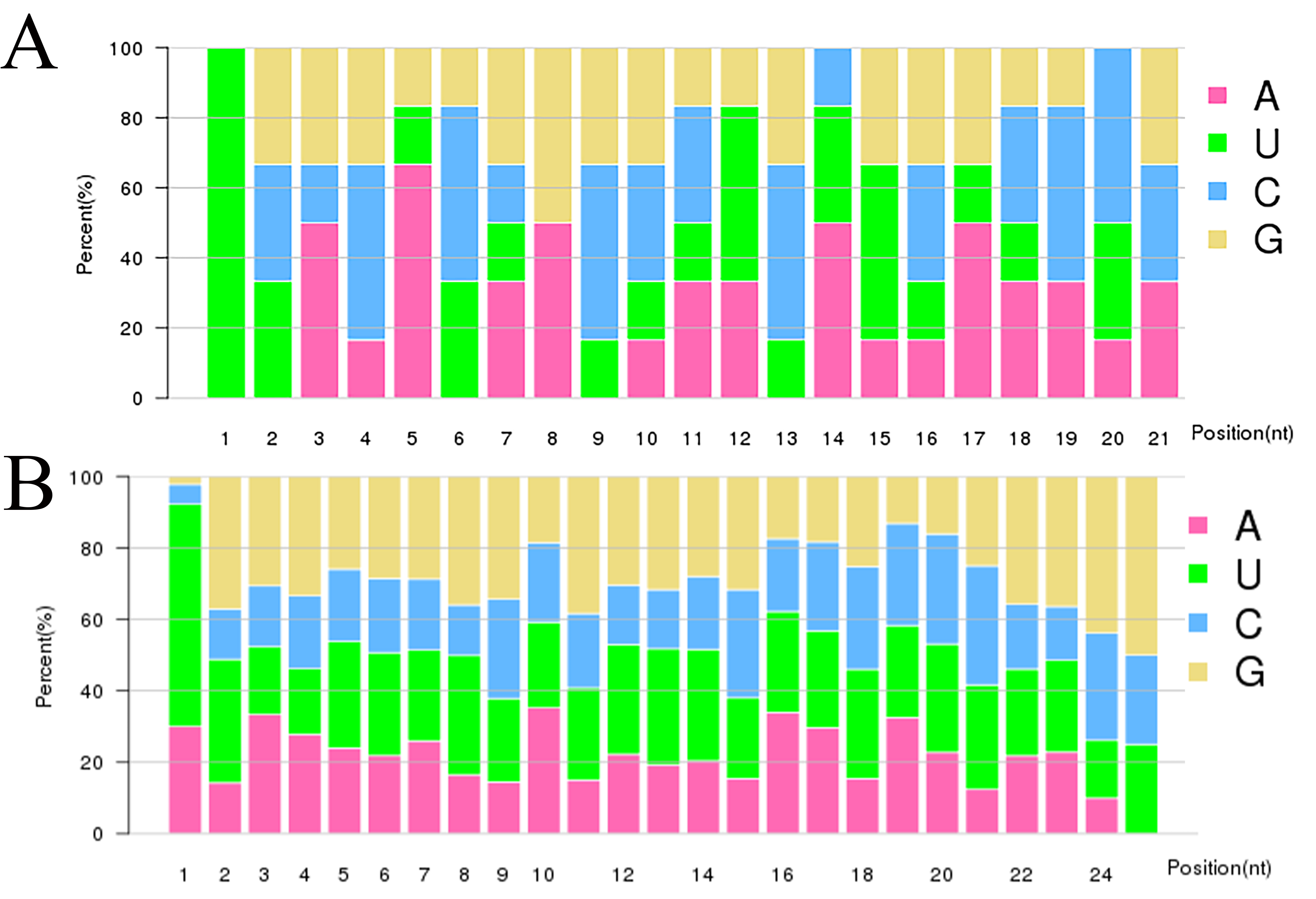

Supplement: Supplementary Figure 5 — Base preferences for miRNA. (A) Base bias of known miRNAs. (B) Base bias of novel miRNAs. The abscissa is the base position of miRNA while the ordinate is the percentage of bases A/U/C/G appearing in the miRNA at that position. [file Image_5.TIF]
